# A New Model for Predicting Hypothyroidism After Intensity-Modulated Radiotherapy for Nasopharyngeal Carcinoma

**DOI:** 10.3389/fonc.2020.551255

**Published:** 2020-09-25

**Authors:** Liang Peng, Yan-Ping Mao, Cheng-Long Huang, Rui Guo, Jun Ma, Wei-Ping Wen, Ling-Long Tang

**Affiliations:** ^1^Department of Radiation Oncology, Sun Yat-sen University Cancer Center, State Key Laboratory of Oncology in South China, Collaborative Innovation Center for Cancer Medicine, Guangzhou, China; ^2^Guangdong Key Laboratory of Nasopharyngeal Carcinoma Diagnosis and Therapy, Guangzhou, China; ^3^Department of Otorhinolaryngology Head and Neck Surgery, The First Affiliated Hospital of Sun Yat-sen University, Guangzhou, China

**Keywords:** nasopharyngeal carcinoma, hypothyroidism, intensity-modulated radiotherapy, dosimetry parameters, predicting model

## Abstract

**Objectives:**

To develop a model that can predict the risk of hypothyroidism (HT) after intensity-modulated radiotherapy (IMRT) for nasopharyngeal carcinoma (NPC), and to accordingly recommend dose constraints.

**Materials and Methods:**

NPC patients treated between 2011 and 2015 were retrospectively reviewed. HT was defined by an abnormally high level of thyrotropin. The dosimetry parameters V_x_ (percentage of thyroid volume receiving more than x Gy of radiation) and V_a,b_ (percentage of thyroid volume receiving >a Gy, while ≤b Gy radiation) were calculated. The primary endpoint was the development of HT within the first 2 years after IMRT. The least absolute shrinkage and selection operator and multivariate logistic regression were used to identify predictors of HT.

**Results:**

A total of 545 patients were included in the analyses, with a median follow-up of 36 months. Of the 545 patients, 138 developed HT within 2 years, and the 2-year incidence of HT was 25.3%. In patients with thyroid volume >20 cm^3^, the 2-year incidence of HT was 11.7% (16/137); in patients with thyroid volume ≤20 cm^3^ and V_30_,_60_ ≤ 80%, the 2-year HT incidence was 19.9% (33/166); in patients with thyroid volume ≤20 cm^3^ and V_30_,_60_ > 80%, the 2-year incidence of HT was 36.8% (89/242).

**Conclusion:**

Thyroid volume and V_30_,_60_ could be reliable predictors of HT after IMRT for NPC. For patients with thyroid volume ≤20 cm^3^, thyroid V_30_,_60_ ≤ 80% might be a useful dose constraint to adopt during IMRT planning.

## Introduction

Radiation-induced hypothyroidism (HT) is frequent after radiotherapy (RT) of the head and neck region, with an incidence of 20–50% (1). Nasopharyngeal carcinoma (NPC) tends to metastasize through the lymphatic network to the neck (2); hence, definitive or prophylactic RT of the neck region can affect the thyroid gland and result in HT. The survival of NPC patients has improved significantly after the introduction of chemotherapy and intensity-modulated radiotherapy (IMRT) during the last two decades (3). As quality of life is an important concern in treatment management, the prediction and prevention of HT after RT is critical for NPC patients.

With IMRT, it is possible to achieve sufficient dose coverage of the tumor and improve tumor control. Additionally, IMRT is enabled with precise dose estimates for organs at risk (OARs), as a result of which it is possible to alleviate normal tissue damage by setting dose constraints on OARs (4). The thyroid gland is composed of functional units, namely, follicles, which produce and release thyroid hormones independently. Radiation above a specific threshold dose will damage these follicles, and dysfunction of too many follicles results in HT. Therefore, dosimetry parameter depicting both the threshold dose of follicle and the number of influenced follicles will be a promising predictor for HT after RT, and that is the parameter Vx, defined as the percentage of thyroid volume receiving more than a to-be-determined threshold dose, x Gy. However, so far, there is no consensus on such a threshold dose, and the reported threshold dose ranges from 30 to 50 Gy (5–11). Previous studies have generally selected the best Vx for predicting HT based on multivariate regression analysis (5–8). However, the reported results are limited by the collinearity of the data caused by their overlap: that is, V_10_ would also include V_20_. In order to minimize the influence of collinearity, we have proposed a prediction model based on novel dosimetry parameters which are generated by subtractions between Vx.

The aim of this study is to determine the dosimetry predictors for HT after RT in NPC patients using a new prediction model, and to recommend the dose constraints on the thyroid gland accordingly.

## Materials and Methods

### Patient Selection

We retrospectively reviewed patients with newly diagnosed, biopsy-proven NPC treated at our institution between June 2011 and December 2015. Patients were included if they were identified as euthyroid before RT and were assessed for thyroid function regularly after RT. The exclusion criteria were previous thyroid surgery, pre-existing pituitary disorders, previous irradiation to the head and neck region or the whole body, and absence of thyroid function assessment after RT. The Institutional Review Board and the Ethics Committee of Sun Yat-sen University Cancer Center approved of this study. As this was a retrospective analysis of routine data, the need for written informed consent from the patients was waived. The key raw data used in this article have been uploaded to the Research Data Deposit public platform^1^.

### Treatment

All patients underwent definitive IMRT. The prescribed doses were 66–72 Gy at 2.00–2.43 Gy per fraction to the nasopharynx gross tumor volume (GTVnx), 64–70 Gy to the malignant lymph nodes (GTVnd), 60–63 Gy to the high-risk clinical target volume (CTV1), and 54–56 Gy to the low-risk clinical target volume (CTV2). The prescribed doses were delivered in 28–33 fractions at the rate of 5 fractions per week. Details of the protocol for target volume delineation and dose constraints on OARs have been reported previously in a randomized controlled trial conducted at our institution (12). There was no dose constraint on the thyroid gland when the IMRT was planned. According to the guidelines of our center, RT alone was recommended for patients with stage I disease, and RT plus platinum-based concurrent chemotherapy with or without induction chemotherapy or adjuvant chemotherapy was recommended for patients with stage II–IVA disease.

### Thyroid Dosimetry Parameters and Function Test

Before treatment, a computed tomography (CT) simulation scan, including plain and enhanced CT scans, were taken (slice thickness = 3 mm); the imaging area extended from the top of the head to 2 cm below the sternoclavicular joint. Contouring of the thyroid gland on the CT simulation images was performed with the Monaco treatment planning system (version 5.0, Elekta AB, Stockholm, Sweden). Dose-volume histograms were retrieved from the planning system, including absolute thyroid volume and the percentage of thyroid volume receiving more than 10, 20, 30, 40, 50, and 60 Gy (V_10_, V_20_, V_30_, V_40_, V_50_, and V_60_) of radiation. The percentage of thyroid volume receiving 0–10 Gy (V_0_,_10_) was calculated as 100% minus V_10_; V_10_,_20_, as V_10_ minus V_20_; V_20_,_30_, as V_20_ minus V_30_; V_30_,_40_, as V_30_ minus V_40_; V_40_,_50_, as V_40_ minus V_50_; V_50_,_60_, as V_50_ minus V_60_.

After treatment was completed, the patients were followed up at least every 3 months during the first 2 years, every 6 months for at least the next 3 years, and annually thereafter. The thyroid function test was performed during the follow-up visit. HT was diagnosed if the thyrotropin (TSH) level was higher than the upper limit of our institutional reference range (0.27–4.20 μIU/mL) and the free thyroxine (fT4) level was not higher than the upper limit of our institutional reference range (12.00–22.00 pmol/L), regardless of symptoms.

### Statistical Analysis

Time to HT was calculated from the end of RT to the first appearance of abnormally high levels of TSH, which is indicative of HT. Patients without HT were censored at the date of the last follow-up or death. Time to HT distribution was estimated using the Kaplan–Meier method, and curves were compared using the log-rank test. The primary endpoint of our study was the development of HT within the first 2 years after RT. The least absolute shrinkage and selection operator (LASSO) was used to select dosimetry parameters with the glmnet package in R (13). The parameters that were selected by LASSO were entered into the multivariate logistic regression model through the backward elimination method. The cutoff value of the dosimetry parameter in the final multivariate model was determined using X-tile (14). SPSS version 22.0 (IBM Corporation, Armonk, NY, United States) and R version 3.6.0^2^ were used for statistical analyses. A two-tailed *P*-value of <0.05 was considered to indicate statistical significance.

## Results

### Characteristics of the Patient Cohort

**FIGURE 1:**
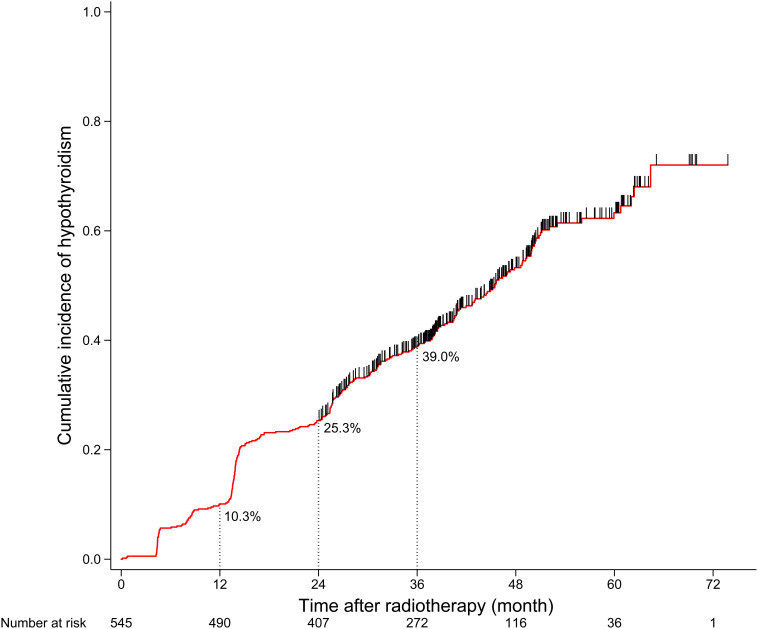
Cumulative incidence curve of hypothyroidism for the whole cohort.

A total of 564 patients met the inclusion criteria, of which 19 patients who were censored within 2 years after RT were excluded from the next analysis. After a median follow-up duration of 36.0 months, 277 of the final cohort of 545 patients developed HT. The 1-, 2-, and 3-year cumulative incidence rates of HT were 10.3, 25.3, and 39.0%, respectively (Figure 1). For 277 patients who developed HT, the median time to HT was 24.2 months; and 138 patients developed HT within 2 years after RT. The clinical and dosimetry characteristics of patients with and without HT within 2 years after RT are summarized in Table 1. The data indicate that female and younger patients are more likely to develop HT within 2 years after RT. Compared to patients without HT, patients with HT had smaller thyroid volumes, higher V_40_,_50_ and lower V_0_,_10_, V_10_,_20_, and V_20_,_30_.

**TABLE 1:** Clinical and dosimetry characteristics in patients with and without HT.

Characteristic	With HT	Without HT	*P-*value
Age (year)	41(33–48)	44(36–52)	0.008^†^
Sex			0.027^‡^
Male	87(63.0%)	297(73.0%)	
Female	51(37.0%)	110(27.0%)	
T stage*			0.174^‡^
T1–2	43(31.2%)	153(37.6%)	
T3–4	95(68.8%)	254(62.4%)	
N stage*			0.064^‡^
N0–1	82(59.4%)	277(68.1%)	
N2–3	56(40.6%)	130(31.9%)	
Overall stage*			0.116^‡^
I–II	28(20.3%)	110(27.0%)	
III–IVA	110(79.7%)	297(73.0%)	
Chemotherapy			0.668^‡^
Yes	121(87.7%)	351(86.2%)	
No	17(12.3%)	56(13.8%)	
Thyroid volume (cm^3^)	14.7(11.6–17.7)	16.4(13.2–20.9)	< 0.001^†^
V_0_,_10_ (%)^§^	0.00(0.00–0.00)^#^	0.00(0.00–0.00)^#^	0.014^†^
V_10_,_20_ (%)	0.00(0.00–0.00)^#^	0.00(0.00–0.20)^#^	0.014^†^
V_20_,_30_ (%)	0.00(0.00–6.41)	0.59(0–10.27)	0.010^†^
V_30_,_40_ (%)	10.25(3.90–19.64)	11.00(5.94–18.93)	0.293^†^
V_40_,_50_ (%)	33.22(23.71–45.02)	28.83(18.28–40.41)	0.011^†^
V_50_,_60_ (%)	33.95(18.21–53.80)	32.12(18.63–45.63)	0.146^†^
Total	138(100%)	407(100%)	

Data presented as median (interquarter range) or number (percentage). HT, hypothyroidism. *According to the 8th edition of the AJCC staging system. ^§^V_*a*,*b*_ represents the percentage of thyroid volume receiving irradiation doses of a–b Gy. ^†^Mann–Whitney U-test. ^‡^Pearson Chi-square test. ^#^Patients without HT have the larger V_0_,_10_ and V_10_,_20_.

### Selection of Dosimetry Parameters

The results of unadjusted univariate logistic regression for dosimetry parameters associated with HT within 2 years after RT are shown in Table 2. Of these dosimetry parameters, thyroid volume, V_10_,_20_ and V_40_,_50_ were statistically significant predictors of HT within 2 years after RT. With the LASSO method, thyroid volume, V_10_,_20_, V_40_,_50_, and V_50_,_60_ were identified, based on logistic regression, as predictors of HT within 2 years after RT. These LASSO-selected parameters were entered into the multivariate logistic regression, with which only thyroid volume was found to be a significant predictor of HT (Table 2). The LASSO-selected parameters were further selected by backward elimination in the multivariate logistic regression model, and thyroid volume and V_40_,_50_ remained as significant predictors (Table 2).

**TABLE 2:** Results of logistic regression for the development of HT.

Variables	Univariate		Multivariate^†^		Multivariate^‡^	
	Odds ratio (95% CI)	*P-*value	Odds ratio (95% CI)	*P-*value	Odds ratio (95% CI)	*P-*value
Thyroid volume (per cm^3^ increase)	0.94 (0.91–0.97)	<0.001	0.94 (0.91–0.97)	0.001	0.94 (0.91–0.97)	<0.001
V_0_,_10_ (per 10% increase)*	0.91 (0.79–1.04)	0.163				
V_10_,_20_ (per 10% increase)	0.66 (0.47–0.93)	0.017	0.82 (0.54–1.26)	0.375		
V_20_,_30_ (per 10% increase)	0.84 (0.65–1.08)	0.172				
V_30_,_40_ (per 10% increase)	0.97 (0.80–1.17)	0.736				
V_40_,_50_ (per 10% increase)	1.16 (1.03–1.32)	0.017	1.15 (0.99–1.33)	0.077	1.17 (1.03–1.33)	0.013
V_50_,_60_ (per 10% increase)	1.09 (0.99–1.20)	0.084	1.06 (0.94–1.19)	0.356		

HT, hypothyroidism; CI, confidence interval. *V_*a*,*b*_ represents the percentage of thyroid volume receiving irradiation doses of a–b Gy. ^†^The LASSO-selected parameters were included. ^‡^Backward elimination with an exclusion criterion of 0.1 was applied to further select the LASSO-selected parameters in the model.

### Inferring the Threshold Dose for the Thyroid

**FIGURE 2:**
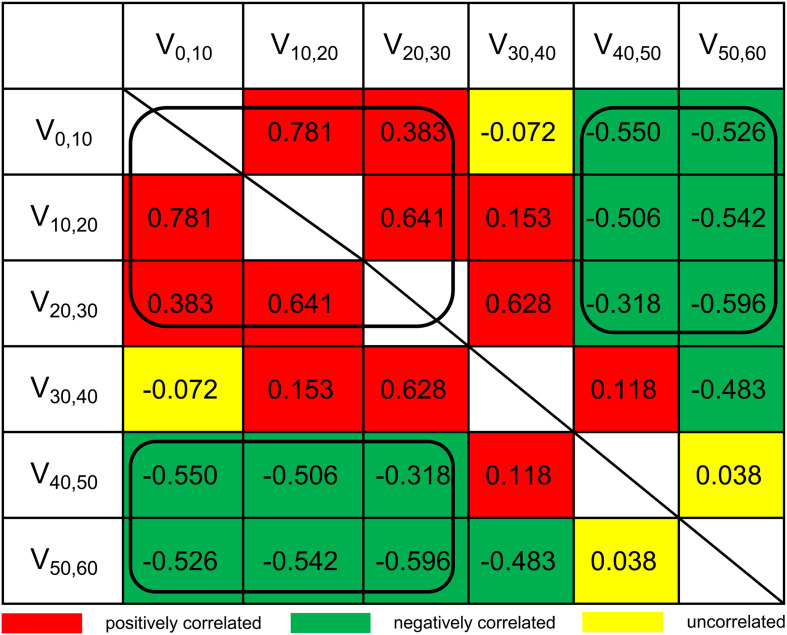
Pairwise Spearman correlation coefficients between dosimetry parameters. V_a,b_, the percentage of thyroid volume receiving irradiation doses of a–b Gy.

Although LASSO was adopted to reduce the effects of collinearity among dosimetry parameters, the correlation among the LASSO-selected parameters cannot be neglected (Figure 2). Obviously, the thyroid volume receiving higher doses of irradiation (V_40_,_50_, V_50_,_60_) would increase if the thyroid volume receiving lower doses (V_0_,_10_, V_10_,_20_, V_20_,_30_) decreased. From the univariate logistic regression analysis shown in Table 2, it was inferred that irradiation doses of 40–50 Gy and above would damage the thyroid gland. This means that V_40_,_50_ and V_50_,_60_ were positive predictors for HT. V_50_,_60_ might not have achieved statistical significance (*P* = 0.084) due to its correlation with the other predictors. Similarly, it was inferred that irradiation doses of 20–30 Gy and below would not have destructive effect on the thyroid gland. However, it was unclear whether doses of 30–40 Gy would damage the thyroid gland. Under the presumption that doses of 30–40 Gy would damage the thyroid gland, V_10_,_20_ and V_50_,_60_ (selected by LASSO) might have been excluded by backward elimination because V_10_,_20_ (negative predictor for HT) correlated positively with V_30_,_40_ (positive predictor for HT), and V_50_,_60_ (positive predictor for HT) correlated negatively with V_30_,_40_ (positive predictor for HT) (Figure 2). To further validate this presumption that the threshold dose that causes damage to the thyroid gland was 30 Gy rather than 40 Gy, we substituted V_30_,_60_ (percentage of thyroid volume receiving doses of 30–60 Gy) or V_40_,_60_ (percentage of thyroid volume receiving doses of 40–60 Gy) with V_40_,_50_ in the final model. Different models were evaluated for their prediction performance based on the area under the curve (AUC). The model that included thyroid volume and V_30_,_60_ had the largest AUC (Table 3).

**TABLE 3:** Different logistic regression models and their area under the curve.

Models	Odds ratio (95% CI)		AUC (95% CI)
	Thyroid volume	V_a,b_* (per 10% increase)	
Model 1 (thyroid volume, V_40_,_50_)	0.94 (0.91–0.97)	1.17 (1.03–1.33)	0.633 (0.582–0.684)
Model 2 (thyroid volume, V_30_,_60_)	0.94 (0.91–0.97)	1.16 (1.05–1.27)	0.643 (0.590–0.695)
Model 3 (thyroid volume, V_40_,_60_)	0.94 (0.91–0.98)	1.12 (1.03–1.22)	0.636 (0.583–0.689)

*V_*a*,*b*_ represents the percentage of thyroid volume receiving irradiation doses of a–b Gy.

### Prediction Model of HT

**FIGURE 3:**
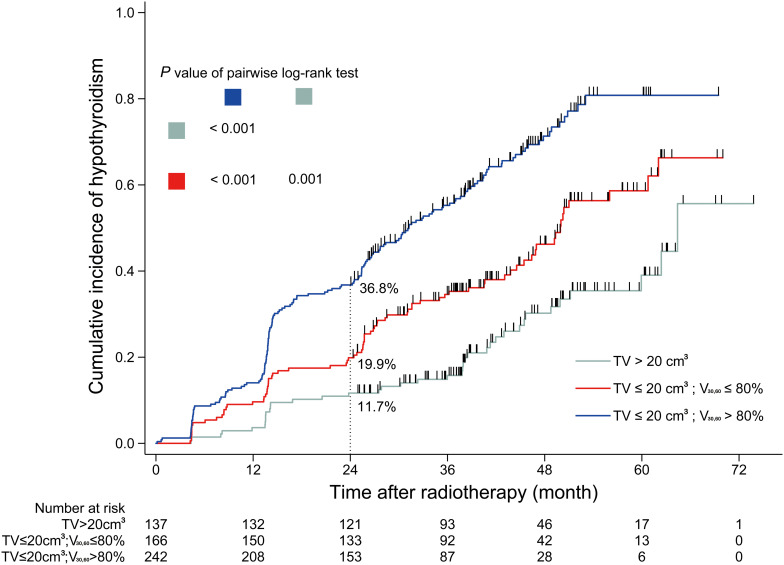
Cumulative incidence curve of hypothyroidism for sub-cohorts. TV, thyroid volume; V_30_,_60_, the percentage of thyroid volume receiving irradiation doses of 30–60 Gy.

Based on the analyses above, a dose constraint on thyroid was set based on thyroid volume and V_30_,_60_. The dichotomous cutoff values determined by X-tile were 20 cm^3^ for thyroid volume and 80% for V_30_,_60_. Patients were grouped into four cohorts, and the incidence rates of HT within 2 years after RT were calculated for each cohort. In cohort 1 comprising patients with thyroid volume >20 cm^3^ and V_30_,_60_ ≤ 80%, the HT incidence rate was 8.0% (4/50). In cohort 2 comprising patients with thyroid volume >20 cm^3^ and V_30_,_60_ > 80%, the HT incidence was 13.8% (12/87). In cohort 3 comprising patients with thyroid volume ≤20 cm^3^ and V_30_,_60_ ≤ 80%, the HT incidence was 19.9% (33/166). Finally, in cohort 4 comprising patients with thyroid volume ≤20 cm^3^ and V_30_,_60_ > 80%, the HT incidence rate was 36.8% (89/242). Cohort 1 and 2 were combined because of the similar incidence rates of HT within 2 years after RT. The cumulative incidence curves of HT in different cohorts during the entire follow-up period are shown in Figure 3.

## Discussion

In the current study, we have investigated the incidence of radiation-induced HT in a large cohort of NPC patients who underwent IMRT, with the aim of determining the dosimetry parameters that could be used as predictors of HT. The 2-year incidence of HT in the present study cohort was 25.3%. The HT defined in this study was actually biochemical HT or subclinical HT, which was diagnosed based on the thyroid function test, regardless of symptoms (15). Although only 2.4–4.3% of patients with subclinical HT progress to overt HT each year (16), subclinical HT is associated with an increased risk of cardiovascular and cerebrovascular morbidity and mortality in younger patients (under the age of 65 years) and in those with high TSH levels (17, 18). For high-risk patients with subclinical HT, the guidelines recommend treatment with levothyroxine (19). Thus, it is of clinical importance to identify the predictors of radiation-induced HT and to reduce its incidence in NPC patients receiving RT.

Although the parameter V_x_ can depict the dose distribution of thyroid well, there is repeated information among different V_x_. For example, V_10_ consists of V_20_ and V_10_,_20_, which would result in the problem of collinearity during statistical analysis. In the current study, we were able to reduce collinearity among dosimetry parameters to some extent by new dosimetry parameter V_a,b_ (the percentage of thyroid volume receiving a–b Gy) which was generated from subtractions between V_x_. Based on our analysis, we propose 30 Gy to be the threshold dose, and dose above 30 Gy is likely to cause damage to the thyroid follicles and lead to deficiency in thyroid function.

Apart from V_30_,_60_, the absolute thyroid volume was also identified as an independent predictor of HT in our analysis. This is probably because the absolute thyroid volume represents the functional reserve of thyroid. The thyroid volume data of this cohort follows normal distribution (Supplementary Figure 1), with a mean value similar to a Korea normal cohort (20). Considering above, thyroid volume data in this study should not be biased. However, the thyroid volume is influenced by sex, age, and body size, and may differ across different populations (20, 21). When generalizing our model to a different population, the cut-off value of thyroid volume should be reconsidered. The thyroid damage model proposed by our study is simple as it only considers the direct radiation-induced damage to independent thyroid functional units. However, radiation-induced damage to thyroid vessels and immune-mediated damage may also influence the thyroid (22). We are aware that a prediction model generated from clinical data may not be able to explain all the biological processes perfectly. It is its clinical utility that makes it a valuable prediction model, as patients at different levels of risk of HT can be well differentiated based on thyroid volume and V_30_,_60_.

We determined the cut-off value of thyroid volume and V_30_,_60_ using X-tile method, which is based on choosing the lowest *P*-value, which might exaggerate the performance of our model. Thus, the performance of our model should be further validated in another cohort.

This study is limited by a potential selection bias, as only patients who underwent regularly thyroid function tests were included: that is, patients who had a better prognosis and stayed alive for a longer time were more likely to be included. To rule out the possibility of severe selection bias, we compared the characteristics of NPC patients treated during the same period in our institution and characteristics of patients included in our study (Supplementary Table 1). We found that cohort of our study had younger age than the reference cohort. Considering above, age should be taken into consideration when generalizing our prediction model. Finally, in order to overcome the follow-up bias that patients with longer follow-up had more chances of developing HT, the primary endpoint was defined as the development of HT within the first 2 years after RT, and patients who were censored within 2 years were excluded from analysis.

## Conclusion

In conclusion, the results of this study indicate that the combination of thyroid volume and V_30_,_60_ could be useful as a predictor of the risk of HT within 2 years after RT in NPC patients who undergo IMRT. In patients with a greater thyroid volume (>20 cm^3^), the radiation dose distribution did not seem to have a considerable influence on the thyroid gland (the 2-year incidence of HT was as low as about 10%). However, for patients with smaller thyroid volume (≤20 cm^3^), a dose constraint of V_30_,_60_ on ≤80% of the thyroid volume should be applied without compromising tumor coverage. Besides, further validation studies using an independent cohort are warranted to compare the performance of our model and other similar models.

## Data Availability Statement

The datasets presented in this study can be found in online repositories. The names of the repository/repositories and accession number(s) can be found at: www.researchdata. org.cn (approval number RDDA2019001022).

## Ethics Statement

The studies involving human participants were reviewed and approved by the Institutional Review Board and the Ethics Committee of Sun Yat-sen University Cancer Center. Written informed consent for participation was not required for this study in accordance with the national legislation and the institutional requirements.

## Author Contributions

L-LT, LP, and C-LH: conceptualization. LP, Y-PM, and C-LH: methodology, investigation, and data curation. LP and C-LH: software and formal analysis. LP, C-LH, and RG: validation. L-LT and JM: resources. LP, C-LH, RG, and Y-PM: writing – original draft preparation. W-PW, Y-PM, JM, and L-LT: writing – review and editing. L-LT: supervision, project administration, and funding acquisition. All authors contributed to the article and approved the submitted version.

## Conflict of Interest

The authors declare that the research was conducted in the absence of any commercial or financial relationships that could be construed as a potential conflict of interest.

## Footnotes

1 www.researchdata.org.cn, approval number RDDA2019001022

2 http://www.rproject.org/

## Supplementary Material

Supplementary Figure 1 The distribution of thyroid volume of the cohort.
