# Supplementary material for: A New Model for Predicting Hypothyroidism After Intensity-Modulated Radiotherapy for Nasopharyngeal Carcinoma
Source: Front Oncol. 2020 Sep 25;10:551255. doi: 10.3389/fonc.2020.551255 (PMC7546200; doi:10.3389/fonc.2020.551255)
Supplement: Supplementary file 1 [file Table_1.DOCX]

**Supplementary Table S1.** Comparison of characteristics between study cohort and reference cohort

| **Characteristic** | **Study cohort (N=545)** | **Reference cohort (N=9155)** | **P-value** |
| --- | --- | --- | --- |
| Age, median and IQR | 42 (35-51) | 45 (38-53) | < 0.001 |
| Sex |  |  | 0.149 |
| Male | 384 (70.5%) | 6714 (73.3%) |  |
| Female | 161 (29.5%) | 2441 (26.7%) |  |
| T stage |  |  | 0.419 |
| T1 | 103 (18.9%) | 1509 (16.5%) |  |
| T2 | 93 (17.1%) | 1487 (16.2%) |  |
| T3 | 241 (44.2%) | 4242 (46.3%) |  |
| T4 | 108 (19.8%) | 1917 (20.9%) |  |
| N stage |  |  | 0.939 |
| N0 | 84 (15.4%) | 1438 (15.7%) |  |
| N1 | 275 (50.5%) | 4558 (49.8%) |  |
| N2 | 115 (21.1%) | 2017 (22.0%) |  |
| N3 | 71 (13.0%) | 1142 (12.5%) |  |
| Overall Stage |  |  | 0.489 |
| I | 38 (7.0%) | 509 (5.6%) |  |
| II | 100 (18.3%) | 1598 (17.5%) |  |
| III | 242 (44.4%) | 4213 (46.0%) |  |
| IVA | 165 (30.3%) | 2835 (31.0%) |  |
| Chemotherapy |  |  | 0.055 |
| Yes | 472 (86.6%) | 8178 (89.3%) |  |
| No | 73 (13.4%) | 977 (10.7%) |  |

| Female | 161 (29.5%) | 2441 (26.7%) |
| --- | --- | --- |
| T stage | | 0.419 |
| T1 | 103 (18.9%) | 1509 (16.5%) |
| T2 | 93 (17.1%) | 1487 (16.2%) |
| T3 | 241 (44.2%) | 4242 (46.3%) |
| T4 | 108 (19.8%) | 1917 (20.9%) |
| N stage | | 0.939 |
| N0 | 84 (15.4%) | 1438 (15.7%) |
| N1 | 275 (50.5%) | 4558 (49.8%) |
| N2 | 115 (21.1%) | 2017 (22.0%) |
| N3 | 71 (13.0%) | 1142 (12.5%) |
| Overall Stage | | 0.489 |
| I | 38 (7.0%) | 509 (5.6%) |
| II | 100 (18.3%) | 1598 (17.5%) |
| III | 242 (44.4%) | 4213 (46.0%) |
| IVA | 165 (30.3%) | 2835 (31.0%) |
| Chemotherapy | | 0.055 |
| Yes | 472 (86.6%) | 8178 (89.3%) |
| No | 73 (13.4%) | 977 (10.7%) |
