# Supplementary figures and images for: A New Model for Predicting Hypothyroidism After Intensity-Modulated Radiotherapy for Nasopharyngeal Carcinoma
Source: Front Oncol. 2020 Sep 25;10:551255. doi: 10.3389/fonc.2020.551255 (PMC7546200; doi:10.3389/fonc.2020.551255)

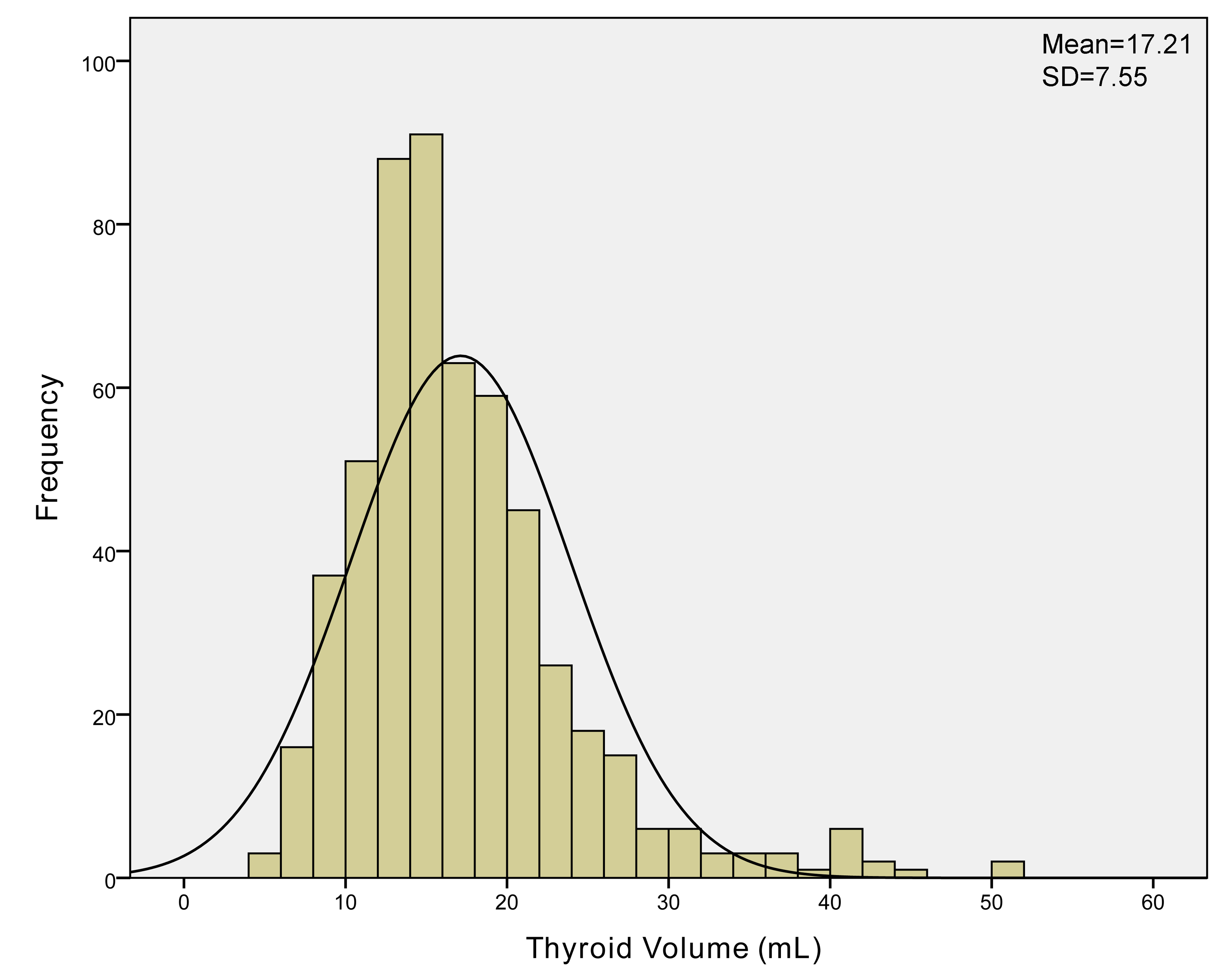

Supplement: Supplementary Figure 1 — The distribution of thyroid volume of the cohort. [file Image_1.TIF]
